# Osteoprotegerin and TRAIL in Acute Onset of Atrial Fibrillation

**DOI:** 10.1155/2015/259843

**Published:** 2015-10-04

**Authors:** Krzysztof Rewiuk, Tomasz Grodzicki

**Affiliations:** Chair of Internal Medicine and Gerontology, Jagiellonian University Medical College, Ulica Śniadeckich 10, 31-531 Kraków, Poland

## Abstract

*Background*. There is a growing amount of evidence that inflammatory processes are involved in the development of atrial fibrillation (AF) and its complications. We decided to investigate the behavior of osteoprotegerin (OPG) and TNF-related apoptosis inducing ligand (TRAIL) in terms of acute onset of AF. *Methods and Results*. We included 60 patients with acute onset of AF, candidates for pharmacological cardioversion. The presence of cardiovascular comorbidities was connected with higher concentration of OPG and lower level of TRAIL right from the first hours of AF paroxysm. The initial TRAIL level correlated also positively with left ventricle ejection fraction and negatively with left atrium diameter. We found subsequent increase of OPG in subgroups selected on the basis of CHA_2_DS_2_-VASc scoring. Although basal concentrations of studied markers did not allow prediction of the restoration of sinus rhythm, we observed important increase of TRAIL concentration in subgroup with sinus rhythm maintenance (94.11 ± 29.46 versus 111.39 ± 30.23 pg/mL; *p* = 0.002). *Conclusions*. OPG and TRAIL are associated with the underlying cardiovascular damage in AF, but their balance is modulated by the fact of sinus rhythm restoration. Determining the suitability of OPG and TRAIL as predictive markers in AF requires further prospective studies.

## 1. Introduction

Atrial fibrillation (AF) is the most common clinically significant arrhythmia, and its incidence is still increasing due to the prolongation of life of the general population and improvement of the prognosis of patients with structural heart disease predisposing to the rhythm disturbances [1]. Key research from the turn of the centuries has confirmed that the essential for the prognosis of patients is not so much arrhythmia itself, but rather the associated risk of thromboembolic events [2]. As this risk is generally independent of the pattern of AF (paroxysmal or persistent) it seems to be a legitimate claim that reasons of hypercoagulability in AF are not only hemodynamic. The most widely accepted theory refers to the concept of Virchow's triad of the role of serum factors and endothelial function in the pathogenesis of AF [3]. Among hundreds of analyzed molecules important significance is also attributed to the markers of inflammation, including superfamily of tumor necrosis factor (TNF) receptors.

One of the representatives of this group is osteoprotegerin (OPG), protein playing the key role in the bone homeostasis and binding bone metabolism with calcification processes of the cardiovascular system [4]. Although OPG is not regarded as a classic marker of endothelial function it should be noted that the substance is stored in the Weibel-Palade bodies and secreted simultaneously with von Willebrand factor [5]. OPG in vitro has protective effects on endothelium: it prevents apoptosis of endothelial cells, induces their growth and differentiation, and inhibits further calcification of media [6–8]. Paradoxically, in clinical studies higher concentrations of OPG in serum correlate with the endothelial dysfunction, degree of vessel wall calcification, presence of cardiovascular risk factors, and cardiovascular mortality [8–14]. This phenomenon might be explained by a compensatory increase in the concentration of OPG in response to the endothelium-damaging factors.

Influence of the OPG on the endothelium may be modulated by interaction with TNF-related apoptosis inducing ligand (TRAIL). TRAIL originally identified as a molecule participating in the process of cytotoxicity with respect to tumor cells has been also proved to be involved in a variety of other mechanisms engaged in activation, migration, and proliferation of various cell types. TRAIL's various interference with the particular types of cells is due to its ability to bind to the five different types of receptors (including OPG) [4]. There are observations which indicate a protective effect of the soluble form of TRAIL in the cardiovascular diseases, which could be explained by the resistance of the endothelium against induced apoptosis [7, 15, 16]. It seems that the paradoxical beneficial effect of TRAIL on the endothelium may correspond to the interaction between OPG and TRAIL.

Although there are several pieces of data on the activity of OPG and TRAIL in permanent AF in patients undergoing surgery for mitral valve as well as patients treated with electrical cardioversion [17–20], to our best knowledge this issue has not been studied in subjects with acute onset of AF who underwent pharmacological cardioversion. Therefore, we have undertaken to measure the concentration of both mentioned above markers in plasma of patients with symptomatic AF lasting less than 48 hours and assess its clinical, echocardiographic, and biochemical correlations in this acute state, as well as prognostic significance regarding the presence of sinus rhythm 7–10 days after attempt of pharmacological cardioversion. What is more we explore the dynamics of OPG and TRAIL concentration in the case of sinus rhythm return or AF persistence.

## 2. Materials and Methods

### 2.1. Patients

The study has enrolled consecutive adult patients of the Clinical Department of Internal Diseases and Geriatrics, University Hospital in Cracow, who meet the following criteria: onset of AF lasting less than 48 hours with symptoms that can be attributed unambiguously to the arrhythmia, qualification for pharmacological cardioversion (hemodynamic stability, absence of coronary pain, the chances of maintaining sinus rhythm in the opinion of the treating physician, lack of contraindications to propafenone or amiodarone, and the patient's consent), and informed consent to participate in the study. Exclusion criteria were as follows: hemodynamic instability, acute coronary syndrome, the presence of other indications for electrical cardioversion, acute or chronic inflammatory disease, and cancer.

All study patients were examined twice: during the onset of AF between 12 and 48 hours of the arrhythmia and 7–10 days after the first test.

The treatments used in the meantime including the type and dose of antiarrhythmic drugs were left at the discretion of leading physicians. The participants were divided into two groups based on the maintenance of sinus rhythm or AF persistence during follow-up (SR group: sinus rhythm, AF group: permanent atrial fibrillation).

The fact of atrial fibrillation was confirmed by standard 12-lead ECG. The time of occurrence of arrhythmia was based on the interview with the patient, taking into consideration symptoms, which can be probably associated with the arrhythmia (palpitations, anxiety in the precordial area, etc.).

During the control visit the presence of AF or sinus rhythm was verified by standard 12-lead ECG. The obtained data was supplemented by information on the treatment used in the meantime.

### 2.2. Blood Sample Collection

Blood samples were drawn from antecubital veins into EDTA tubes from all patients in the fasted state. Lipid profile and high sensitive CRP were measured using well-established methods routinely used in clinical practice. For the osteoprotegerin and TRAIL measurement samples taken were centrifuged for 15 minutes at 3500 rpm. The resulting supernatant was frozen at −70°C until analysis. After collection a set of samples have been thawed in the Department of Diagnostic SU in Krakow and used to determine the concentration of both markers.

### 2.3. Enzyme Immunoassays

Plasma humans concentration of osteoprotegerin (BioVendor, Minneapolis, MN, USA) and TRAIL (R&D Systems Research Diagnostic, Brno, Czech Republic) were measured using commercially available ELISA kit. Each measurement was performed according to the manufacturer's instructions.

### 2.4. Echocardiography

All patients underwent two-dimensional transthoracic Doppler echocardiography using a GE Vivid 3 Ultrasound system (General Electric Company) according to the standards of the Polish Cardiac Society. The size of the left atrium was assessed in the parasternal long axis view. For the evaluation of left ventricular ejection fraction (EF) Simpson method was used.

### 2.5. Statistical Analysis

All statistical analyses of the results were performed with Statistica 10 (StatSoft Inc.) measurement. Data are expressed as mean ± SD for normal distribution or medians and ranges for distribution differ from the normal after verification of distribution by Shapiro-Wilk test. For further analyses concentration of OPG was logarithmized. Comparisons of the two independent groups were performed using Student's* t*-test or nonparametric Mann-Whitney test, according to the distribution of variables. To assess the differences between pairs of biochemical measurements (visit I versus visit II)* t*-test for dependent values was used, separately in patients with restored sinus rhythm and atrial fibrillation. The linear correlations between OPG, TRAIL, and continuous variables were analyzed, using Pearson linear correlation coefficient. A *p* value < 0.05 was considered significant.

## 3. Results

### 3.1. Basic Clinical Characteristics of Patients

The study group consisted of 60 patients aged from 26 to 88 years, and mean age was 65.15 ± 10.76 years. Among the respondents, there were 27 women (45% of patients). Average body mass index was 27.27 ± 4.60 kg/m^2^. Among the typical risk factors for atrial fibrillation 50 (83.33%) patients presented with hypertension, 17 (28.33%) with heart failure, 34 (56.67%) with coronary artery disease (including previous myocardial infarction in 8 patients), and 15 with (25%) significant mitral valve disease. Only 4 participants presented no risk factors for atrial fibrillation and were considered as patients with lone form of arrhythmia. The oral prophylaxis with acenocoumarol was realized in 36 (60%) patients. During the second test after 8.42 ± 1.38 days in 39 (65%) subjects sinus rhythm was observed. There were no significant differences between the treatments used in subgroups with presence of sinus rhythm and AF persistence. Comparison of both groups is presented in Table 1.

### 3.2. Initial OPG and TRAIL Concentration and Its Correlations

**Figure 1:**
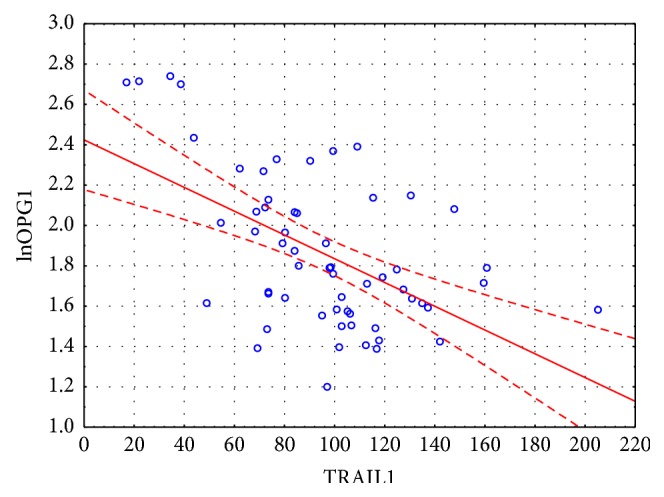
Correlation between initial concentrations of OPG and TRAIL.

**Figure 2:**
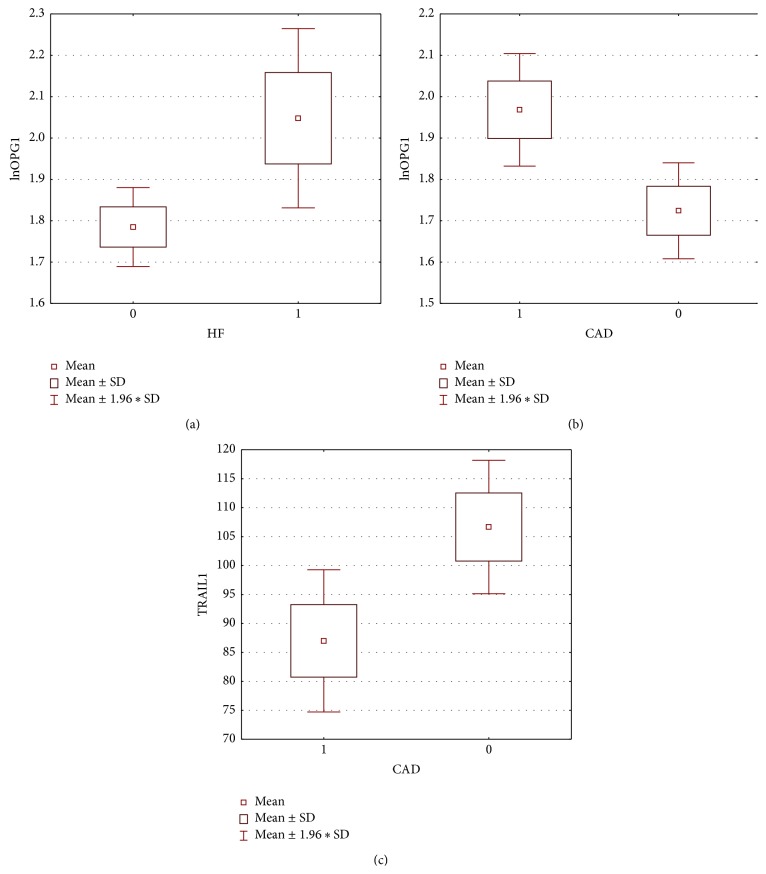
Differences in OPG and TRAIL concentration concerning CAD and HF presence (1: present, 0: absent).

**Figure 3:**
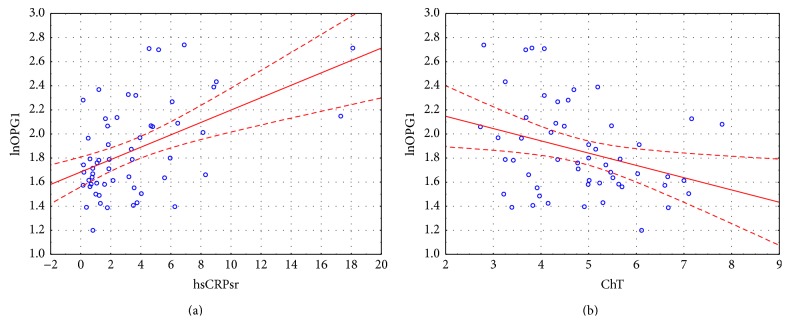
Correlations between initial OPG concentration and hsCRP and total cholesterol.

**Figure 4:**
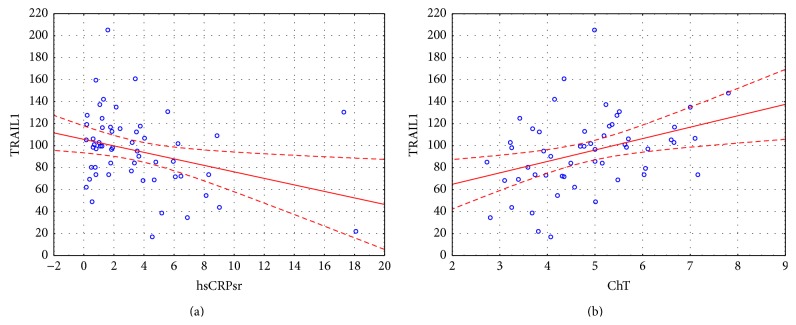
Correlations between initial TRAIL concentration and hsCRP and total cholesterol.

**Figure 5:**
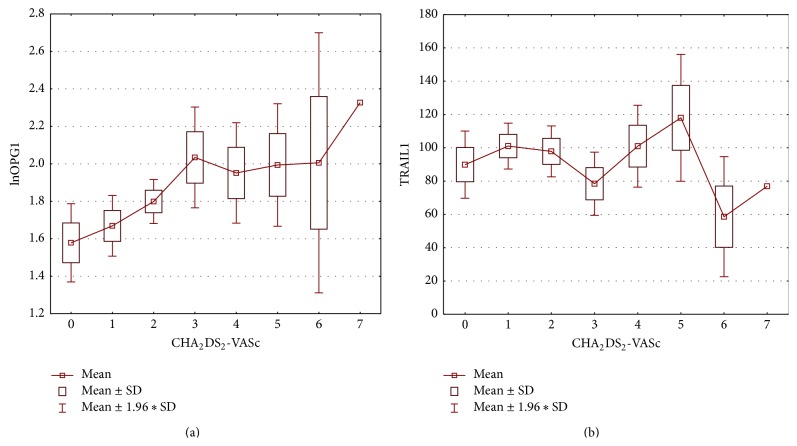
Distribution of OPG and TRAIL depending on CHA_2_DS_2_-VASc score.

The concentration of OPG during first visit (acute onset of AF) was 5.94 [4.86; 8.08] pmol/L, at the control visit 6.40 [5.60; 7.09] pmol/L. The mean concentration of TRAIL was 95.66 ± 34.62 pg/mL and 101.52 ± 32.27 pg/mL, respectively. We found important statistical negative correlation between OPG and TRAIL concentrations at first visit (*r* = −0.54, *p* < 0.001) and weaker but still important one at the control visit (*r* = −0.35, *p* = 0.02) (Figure 1). Important differences between initial concentrations of analyzed markers were observed in subgroups selected on the basis of coronary artery disease (for both markers) and heart failure (for OPG only) presence (Figure 2). The initial level of OPG but not TRAIL correlated with age of patients (*r* = 0.44, *p* < 0.001). We also found correlations between both markers' initial levels and total cholesterol and hsCRP, with opposite direction of mentioned correlations for OPG and TRAIL (Figures 3 and 4). The TRAIL level correlated positively with EF (*r* = 0.33; *p* = 0.01) and negatively with left atrium diameter (*r* = −0.28; *p* = 0.03). The OPG concentration was also higher in patients treated with acetylsalicylic acid (ASA) (lnOPG: 1.97 ± 0.42 versus 1.76 ± 0.31 pmol/L; *p* < 0.05), diuretics (2.06 ± 0.42 versus 1.75 ± 0.31 pmol/L; *p* < 0.01), and insulin (1.84 ± 0.37 versus 2.39 ± 0.29 pmol/L; *p* < 0.01). TRAIL level was higher in patients taking statins (102.07 ± 33.92 versus 83.39 ± 32.33 pg/mL; *p* < 0.05). We have found no other differences in the concentrations of the tested substances depending on the used treatment (including oral antithrombotics). Then we compared initial concentrations of OPG and TRAIL in subgroups selected on the basis of CHA_2_DS_2_-VASc scoring. We found subsequent increase of OPG but not the TRAIL in the model (Figure 5). We also compare OPG level in patients with CHA_2_DS_2_-VASc score <2 and ≥2 pkt. There was important difference in OPG concentration between the subgroups (1.94 ± 0.38 versus 1.65 ± 0.29 pmol/L; *p* < 0.01).

### 3.3. Return of Sinus Rhythm and Changes in Concentrations of OPG and TRAIL

**Figure 6:**
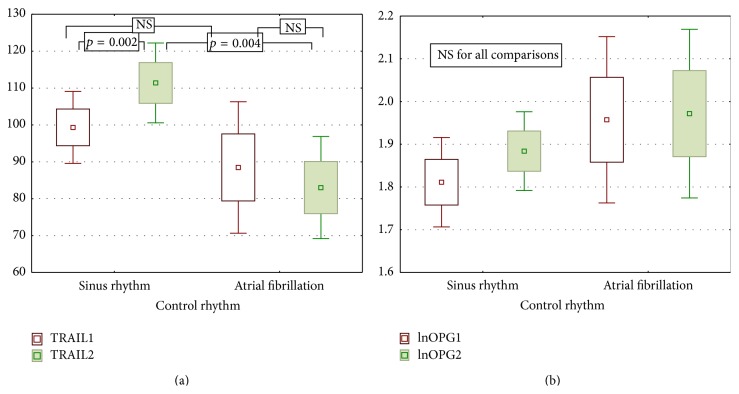
Dynamics of OPG and TRAIL in SR and AF groups.

To evaluate the usefulness of examined markers in predicting the effectiveness of pharmacological cardioversion we compared initial concentrations of OPG and TRAIL in subgroups with SR or AF in control visit. There was no important difference in initial OPG and TRAIL concentration between patients who later restore sinus rhythm or maintain AF. Then, we assessed the dynamics of changes in concentration of both markers in two subgroups separately and we observed important increase in TRAIL concentration in subgroup with SR return (visit I versus visit II: 94.11 ± 29.46 versus 111.39 ± 30.23 pg/mL; *p* = 0.002) and no significant changes in the group of AF maintenance (88.49 ± 40.65 versus 83.03 ± 28.25 pg/mL; NS). At the control visit there was also important difference in the concentration of TRAIL between patients with sinus rhythm and atrial fibrillation (111.39 ± 30.23 versus 83.03 ± 28.25 pg/mL; *p* = 0.004). The level of OPG remained unchanged regardless of the control rhythm (Figure 6).

## 4. Discussion

By assessing the circulating levels of OPG and TRAIL in the patients with acute onset of AF and after pharmacological cardioversion attempt we have demonstrated that the markers present opposite interrelation. These findings could be partially explained by opposite correlations of mentioned markers with hsCRP and total cholesterol levels. Moreover, the results may suggest generally beneficial effects of TRAIL and unfavorable effects of OPG in the mentioned group, as demonstrated particularly by correlation between OPG level and CHA_2_DS_2_-VASc score and change in the concentration of TRAIL in the successful cardioversion subgroup. Although TRAIL and OPG were not useful in predicting the effectiveness of pharmacological cardioversion, defined as the presence of sinus rhythm within 7–10 days after the taking treatment, TRAIL level increased after SR restoration while OPG remained unchanged.

No particular restrictive inclusion criteria caused the study group to reflect the complex spectrum of patients with AF from patients with lone atrial fibrillation to the patients with organic heart damage and numerous comorbidities. Generally, although the sample characteristics were very diverse, its demographic profile, comorbidity, and echocardiographic parameters did not differ considerably from data from large registries for atrial fibrillation [21, 22]. Also, the fact of pharmacological cardioversion choice as a treatment corresponds to the practice of real life. Most studies of the dynamics of biochemical markers in patients with acute AF assess these markers change in demand on the effectiveness of electrical cardioversion. However, pharmacological cardioversion is still the most common method of restoring sinus rhythm in clinical practice [23].

We have a very small amount of data concerning the relationship between the AF and OPG/TRAIL system. The first study which drew attention to the above problem was Schnabel et al.'s work based on the population of the Framingham Offspring Study. Among the tested 12 inflammatory markers only the OPG had significant prognostic value in predicting the occurrence of AF [24]. In a study of Cao et al., significantly higher expression of OPG in biopsy material from right atrial appendage was found in AF patients compared to patients with sinus rhythm [17, 18]. However, we have not identified in the available literature data concerning the levels of serum OPG in patients with acute onset AF.

The mean concentration of TRAIL in the study group was 95.66 ± 34.62 pg/mL. The literature on the relationship between the concentration of TRAIL and AF is very limited. In a small study (involving 25 participants) of Osmancik et al. on patients undergoing ablation of AF they found a mean concentration of the TRAIL 113.7 ± 29.4 pg/mL in patients with paroxysmal AF and 116.9 ± 30.6 in subjects with persistent AF [19]. The subjects, however, were younger (59.5 ± 8.2 years) and coronary artery disease, significant heart failure, and COPD were the exclusion criteria of the study, which can explain a higher concentration of TRAIL than observed in our study.

The results confirm a positive correlation of OPG and negative correlation of TRAIL with cardiovascular risk factors. In the study we noted increasing with age levels of OPG, higher levels of this marker in subgroups with coronary artery disease (CAD) and heart failure, and lower mean concentration of the TRAIL in CAD. What is more OPG concentration was higher in patients treated with ASA, diuretics, and insulin, which can indirectly indicate a higher concentration of the tested substance in patients with more serious damage to the cardiovascular system. On the other side higher levels of TRAIL in subgroup using statins suggest beneficial influence of this drug class on the studied marker system. A positive correlation between the concentration of TRAIL and the EF and negative correlation with the size of the left atrium were also established. With regard to the above relations it is not surprising that we found a negative correlation between serum concentrations of TRAIL and OPG. Because OPG acts as a soluble receptor for TRAIL, the interrelationships between these two substances merit special attention. There is a hypothesis assuming that the fact of the uptake and binding of TRAIL by OPG is a protective mechanism against apoptosis [25]. Since OPG is released from endothelial cells under the influence of the inflammatory cytokines, this hypothesis seems to be particularly accurate with respect to the biology of endothelial cells. However, there is also the opposite hypothesis put forward by Secchiero et al., assuming originally favorable effect of TRAIL depending on its anti-inflammatory and antiatherosclerotic activity, inhibited by OPG as its decoy receptor [26]. Results obtained by us seem to support this second hypothesis.

It is worth noting that our results relate to the soluble form of TRAIL present in the blood serum. TRAIL bound to the cell surface is characterized by a different biology, and the example is the presence of TRAIL-expressing leukocytes within the atherosclerotic lesions and positive relationship between the degree of its expression and the atherosclerotic plaque instability. It can explain the difference between the results obtained by us and the results of Cao et al., who found a positive correlation between the expression of the gene for OPG and the presence of TRAIL transcript in tissue samples taken from the left atrial appendage in patients with AF [17].

The thesis of the favorable impact of TRAIL in biology AF appears to be confirmed in an established increase in the concentration of this substance in patients who have experienced successful cardioversion. This observation remains however in opposition to the results of Osmancik et al., which recorded decrease in the concentration of this marker in patients undergoing successful ablation of AF at 3 and 6 months after surgery [19]. This fact was interpreted by the authors of the publication as evidence of inhibition of cardiomyocyte apoptosis and subsequent fibrosis of atria. As TRAIL in vivo is mainly protective in relation to cardiovascular effects, this makes the above interpretation into question. The discrepancy of our results with data from the cited study beyond those concerning the examined groups differences can be explained by the invasive nature of the intervention and significantly longer duration of sinus rhythm at the time of the assay.

The protective effect of TRAIL in patients with AF was also proved by the results of Deftereos et al. [20]. These researchers determined the concentration of this marker in the blood collected at the time of catheterization of the coronary sinus and the aorta. The difference of these concentrations was presented as transcardial gradient of TRAIL. The study was performed in patients within 7–9 days after successful electrical cardioversion of AF and the results were related to the risk of AF recurrence within 6 months. Higher gradient of TRAIL indicating the increased intracardiac production of this marker proved to be a negative predictor of arrhythmia recurrence.

Among the obtained data particularly noteworthy are those demonstrating “reverse epidemiology” of total cholesterol in the study group. Negative correlation with OPG and positive correlation with TRAIL seem to be counterintuitive. One of the possible explanations is opposite correlation between total cholesterol and hsCRP. This finding underlines the importance of inflammatory processes in biology of AF even overcoming the classical connection between lipid markers and cardiovascular risk [27].

This study is also the first assessing OPG in the context of CHA_2_DS_2_-VASc calculation. Previous results concerning OPG concentration in terms of issues related with the risk of stroke regarded sinus rhythm patients and were contradictory [28, 29]. Observed differences can be partly explained by the relationship between OPG and the age of the respondents and the presence of CAD and HF. On the other hand, it cannot be excluded that the measurement of OPG concentration can provide added value in the prediction of stroke in patients with AF. Establishing this fact, however, is only possible in eventual prospective studies.

## 5. Conclusions

The presented study supported the thesis that OPG and TRAIL may contribute to pathogenesis of AF as, respectively, negative and positive factor. Both markers had been initially connected with the presence of common risk factors but the return of sinus rhythm modulated their balance. Although they were not useful in predicting the return of sinus rhythm, it seems that they may reflect global cardiovascular burden and correspond to thromboembolic risk. Determining the suitability of OPG and TRAIL in predicting complications of AF requires further prospective studies.

## Figures and Tables

**Table 1:** Characteristics of study group with the division into AF persistent and SR restored subgroups.

	SR	AF	*p*
Age (years), mean ± SD	63.38 ± 11.74	68.43 ± 7.90	0.08
Male, *n* (%)	22 (56.41%)	11 (52.38%)	0.77
BMI (kg/m^2^), mean ± SD	**26.22 ± 4.18**	**29.38 ± 4.78**	**<0.05**
Heart rate (bpm), mean ± SD	99.46 ± 25.71	103.86 ± 25.00	0.53
RRsyst (mmHg), mean ± SD	127.44 ± 15.76	132.14 ± 18.27	0.30
RRdiast (mmHg), mean ± SD	82.82 ± 11.05	83.57 ± 10.14	0.80

LA (mm), mean ± SD	**43.03 ± 6.87**	**51.14 ± 7.11**	**<0.001**
Ao (mm), mean ± SD	31.61 ± 4.49	33.86 ± 4.59	0.08
ACS (mm), mean ± SD	19.60 ± 3.21	19.67 ± 2.35	0.94
LVESd (mm), Me [Q1; Q3]	**32.5** ** [30; 36]**	**38 [32; 44]**	**0.01**
LVEDd (mm), Me [Q1; Q3]	**48 [46; 55]**	**54 [50; 56]**	**0.03**
EF (%), Me [Q1; Q3]	**60 [56; 65]**	**51 [35; 61]**	**0.03**
IVSs (mm), mean ± SD	15.26 ± 2.34	14.81 ± 2.89	0.53
IVSd (mm), mean ± SD	11.17 ± 1.62	11.48 ± 2.46	0.58
PWs (mm), Me [Q1; Q3]	16 [14; 17]	16 [14; 18]	0.75
PWd (mm), Me [Q1; Q3]	11 [10; 12]	11 [10; 12]	0.66
RV (mm), Me [Q1; Q3]	24 [22.5; 25]	25 [24; 26]	0.25
TP (mm), Me [Q1; Q3]	24 [22; 25.5]	24 [22; 26]	0.45

Cholesterol tot. (mmol/L), mean ± SD	**5.12 ± 1.32**	**4.22 ± 0.72**	**0.007**
LDL chol. (mmol/L), Me [Q1; Q3]	**3.10 [2.10; 3.50]**	**2.74** ** [1.76; 2.90]**	**0.03**
HDL chol. (mmol/L), Me [Q1; Q3]	**1.42 [1.17; 1.65]**	**1.11 [0.92; 1.21]**	**0.004**
Triglycerides (mmol/L), Me [Q1; Q3]	1.21 [0.95; 1.98]	1.42 [0.92; 2.09]	0.99
hsCRP (mg/L), Me [Q1; Q3]	**1.31 [0.80; 3.43]**	**4.47 [2.89; 7.32]**	**<0.001**

## References

[1] Ball J., Carrington M. J., McMurray J. J. V., Stewart S. (2013). Atrial fibrillation: profile and burden of an evolving epidemic in the 21st century. *International Journal of Cardiology*.

[2] Wyse D. G., Waldo A. L., DiMarco J. P. (2002). A comparison of rate control and rhythm control in patients with atrial fibrillation. *The New England Journal of Medicine*.

[3] Watson T., Shantsila E., Lip G. Y. (2009). Mechanisms of thrombogenesis in atrial fibrillation: Virchow's triad revisited. *The Lancet*.

[4] Baud'huin M., Duplomb L., Teletchea S. (2013). Osteoprotegerin: multiple partners for multiple functions. *Cytokine and Growth Factor Reviews*.

[5] Zannettino A. C. W., Holding C. A., Diamond P. (2005). Osteoprotegerin (OPG) is localized to the Weibel-Palade bodies of human vascular endothelial cells and is physically associated with von Willebrand factor. *Journal of Cellular Physiology*.

[6] Bennett B. J., Scatena M., Kirk E. A. (2006). Osteoprotegerin inactivation accelerates advanced atherosclerotic lesion progression and calcification in older ApoE^−/−^ mice. *Arteriosclerosis, Thrombosis, and Vascular Biology*.

[7] Corallini F., Rimondi E., Secchiero P. (2008). TRAIL and osteoprotegerin: a role in endothelial physiopathology?. *Frontiers in Bioscience*.

[8] Abedin M., Omland T., Ueland T. (2007). Relation of osteoprotegerin to coronary calcium and aortic plaque (from the Dallas Heart Study). *The American Journal of Cardiology*.

[9] Jono S., Ikari Y., Shioi A. (2002). Serum osteoprotegerin levels are associated with the presence and severity of coronary artery disease. *Circulation*.

[10] Kiechl S., Schett G., Wenning G. (2004). Osteoprotegerin is a risk factor for progressive atherosclerosis and cardiovascular disease. *Circulation*.

[11] Omland T., Ueland T., Jansson A. M. (2008). Circulating osteoprotegerin levels and long-term prognosis in patients with acute coronary syndromes. *Journal of the American College of Cardiology*.

[12] Mogelvang R., Pedersen S. H., Flyvbjerg A. (2012). Comparison of osteoprotegerin to traditional atherosclerotic risk factors and high-sensitivity c-reactive protein for diagnosis of atherosclerosis. *The American Journal of Cardiology*.

[13] Blázquez-Medela A. M., García-Ortiz L., Gómez-Marcos M. A. (2012). Osteoprotegerin is associated with cardiovascular risk in hypertension and/or diabetes. *European Journal of Clinical Investigation*.

[14] Stępień E., Fedak D., Klimeczek P. (2012). Osteoprotegerin, but not osteopontin, as a potential predictor of vascular calcification in normotensive subjects. *Hypertension Research*.

[15] Zauli G., Pandolfi A., Gonelli A. (2003). Tumor necrosis factor-related apoptosis-inducing ligand (TRAIL) sequentially up-regulates nitric oxide and prostanoid production in primary human endothelial cells. *Circulation Research*.

[16] Volpato S., Ferrucci L., Secchiero P. (2011). Association of tumor necrosis factor-related apoptosis-inducing ligand with total and cardiovascular mortality in older adults. *Atherosclerosis*.

[17] Cao H., Wang J., Xi L., Røe O. D., Chen Y., Wang D. (2011). Dysregulated atrial gene expression of osteoprotegerin/receptor activator of nuclear factor-*κ*B (RANK)/RANK ligand axis in the development and progression of atrial fibrillation. *Circulation Journal*.

[18] Cao H., Li Q., Li M. (2013). Osteoprotegerin/RANK/RANKL axis and atrial remodeling in mitral valvular patients with atrial fibrillation. *International Journal of Cardiology*.

[19] Osmancik P., Peroutka Z., Budera P. (2010). Decreased apoptosis following successful ablation of atrial fibrillation. *Cardiology*.

[20] Deftereos S., Giannopoulos G., Kossyvakis C. (2013). Association of post-cardioversion transcardiac concentration gradient of soluble tumor necrosis factor-related apoptosis-inducing ligand (sTRAIL) and inflammatory biomarkers to atrial fibrillation recurrence. *Clinical Biochemistry*.

[21] Nieuwlaat R., Capucci A., Camm A. J. (2005). Atrial fibrillation management: a prospective survey in ESC member countries: the Euro Heart Survey on Atrial Fibrillation. *European Heart Journal*.

[22] Alam M., Bandeali S. J., Shahzad S. A., Lakkis N. (2012). Real-life global survey evaluating patients with atrial fibrillation (REALISE-AF): results of an international observational registry. *Expert Review of Cardiovascular Therapy*.

[23] Pisters R., Nieuwlaat R., Prins M. H. (2012). Clinical correlates of immediate success and outcome at 1-year follow-up of real-world cardioversion of atrial fibrillation: the Euro Heart Survey. *Europace*.

[24] Schnabel R. B., Larson M. G., Yamamoto J. F. (2009). Relation of multiple inflammatory biomarkers to incident atrial fibrillation. *The American Journal of Cardiology*.

[25] Pritzker L. B., Scatena M., Giachelli C. M. (2004). The role of osteoprotegerin and tumor necrosis factor-related apoptosis-inducing ligand in human microvascular endothelial cell survival. *Molecular Biology of the Cell*.

[26] Secchiero P., Corallini F., Beltrami A. P. (2010). An imbalanced OPG/TRAIL ratio is associated to severe acute myocardial infarction. *Atherosclerosis*.

[27] Wu N., Xu B., Xiang Y. (2013). Association of inflammatory factors with occurrence and recurrence of atrial fibrillation: a meta-analysis. *International Journal of Cardiology*.

[28] Nybo M., Johnsen S. P., Dethlefsen C. (2008). Lack of observed association between high plasma osteoprotegerin concentrations and ischemic stroke risk in a healthy population. *Clinical Chemistry*.

[29] Mogelvang R., Haahr-Pedersen S., Bjerre M. (2013). Osteoprotegerin improves risk detection by traditional cardiovascular risk factors and hsCRP. *Heart*.

